# Intratumoral microbiota: implications for cancer progression and treatment

**DOI:** 10.3389/fmicb.2025.1551515

**Published:** 2025-07-28

**Authors:** Zehang Xie, Zhenguo Wu, Yan Liu, Yu Gu, Jiahao Niu, Kun Lv

**Affiliations:** ^1^Central Laboratory, The First Affiliated Hospital of Wannan Medical College, Wuhu, China; ^2^Key Laboratory of Non-Coding RNA Transformation Research of Anhui Higher Education Institutes (Wannan Medical College), Wuhu, China

**Keywords:** intratumoral microbiota, tumorigenesis, therapy for anticancer, biomarker, drug metabolism

## Abstract

The human body has a diverse range of microbiota that influences human physiological processes and alters disease risk, involving cancer. Metagenomic sequencing investigations have revealed that the microbiota is an element of the tumor microenvironment, affecting tumor proliferation and responsiveness to current anticancer treatments. The notion of intratumoral microbiota was subsequently introduced. Intratumoral microorganisms have been identified in kinds of cancer, including pancreatic, colorectal, liver, esophageal, breast, and lung malignancies. Microbiota may inhabit tumor tissues by mucosal breakdown, neighboring tissue migration, and hematogenous spread, influencing the biological behavior of tumors as a significant component of tumor’s microenvironment. The intratumoral microbiota may facilitate the onset and progression of malignancies through DNA mutations, activation of carcinogenic pathways, alteration of anticancer medication metabolism, and commencement of metastasis. This document is to present an overview of intratumoral microbiota, their prevalence and progression in cancer, their detection and therapy, and to evaluate the potential and limitations of research in this domain. We intend to generate ideas for investigating intratumoral microbiota as possible treatment targets and biomarkers for tumor assessment, prognosis, and detection.

## 1 Introduction

Bacteria, viruses, fungus, and other eukaryotic organisms may live in a variety of locations throughout the human body, such as the oral cavity, digestive system, reproductive system, and epidermis (Buchta et al., 2019, Matson et al., 2018, Tanoue et al., 2019). However, technological advancements have shown that previously thought-to-be sterile tissues and organs, such as the lung, breast, liver, pancreas, prostate, and kidney, contain low-biomass microbial populations, paving the way for more study in related domains (Nejman et al., 2020, Wong-Rolle et al., 2021). In recent years, research has shown the existence of bacteria in tumors and their critical significance. Seven forms of cancer and their surrounding normal tissues were investigated, including breast, lung, ovarian, pancreatic, melanoma, osteoma, and brain tumors. Each tumor type was discovered to have a distinct microbiome makeup. Different cancer subtypes have been shown to have distinct microbial makeup (Nejman et al., 2020, Huang et al., 2022, Chai et al., 2023). Furthermore, most bacteria are found inside tumor cells, and the critical function of tumor microorganisms in cancer formation, metastasis, and immunotherapy has been gradually revealed (Chen et al., 2017). Intratumoral bacteria may facilitate cancer development and advancement via mechanisms such as damage to DNA, changes in epigenetics, inflammatory reactions, host immunity regulation, and the activation of carcinogenic pathways or cancer genes (Li C. et al., 2021). As a result, a deeper knowledge of the alterations in the intratumoral microbiota may open up new avenues for cancer disease therapy. This review delves into mechanistic investigations of microbial contributions to carcinogenesis and development, as well as the limitations and future directions of existing research. These findings add to prior research on intratumoral microbes and may lay the theoretical foundation for future cancer therapies that target the intratumoural microbiota.

## 2 Origin of intratumoral microbiota

**FIGURE 1:**
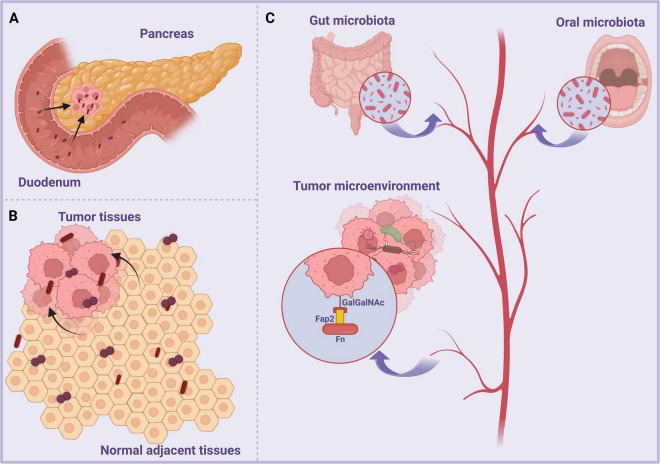
Origins of intratumor microbiota: **(A)** Mucosal organs. Gut microorganisms compromise the mucosal barrier and infiltrate tumor sites, whereas intratumoural bacteria in pancreatic cancer access these sites via the pancreatic duct. **(B)** NATs. NAT may serve as a possible source of intratumoral bacteria. **(C)** Cardiovascular system. Intratumoural microorganisms infiltrate tumor locations from the oral cavity, gastrointestinal tract, and other regions by hematogenous dissemination.

**FIGURE 2:**
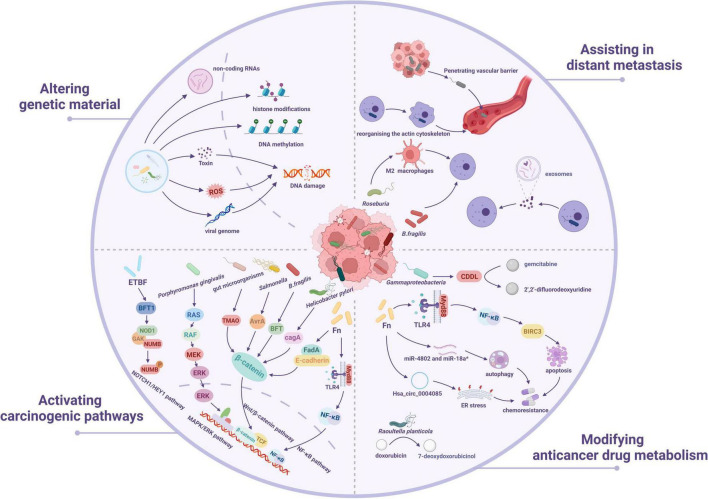
The underlying mechanisms of intratumoral microbiota-mediated cancer development. Intratumor microbiota may play a role in cancer formation through four mechanisms: altering genetic material, activating carcinogenic pathways, modifying anticancer medication metabolism, and facilitating distant metastases.

The tumor microenvironment (TME) is recognized for its vascular growth, aerobic glycolysis, hypoxia, and immunosuppression, making it an ideal home for microbes (Swanton et al., 2024). Despite much focus on the intratumor microbiota, their origins remain inadequately understood. Recent evidence categorizes possible origins of intratumor microbes into three classifications: (1) Intratumoral microbes sourced from mucosal locations across mucosal barrier (Figure 1A), (2) intratumoral microbes arising from normal adjacent tissues (Figure 1B), and (3) intratumor microbes resulting from hematogenous dissemination (Figure 1C; Xie et al., 2022). In several cancer types, the local microbiota is a significant component of the tumor microenvironment, especially in malignancies originating from mucosal regions such as the lungs, skin, and gastrointestinal tract (Wong-Rolle et al., 2021, Ridlon and Gaskins, 2024). In these tumors, tumorigenesis and other factors that compromise the body’s mucosal barriers may facilitate the entry of microorganisms, leading to the establishment of an intratumoral microbiota that may assume complicated functions. Bacteria may potentially originate from adjacent tissues. After examining tumors and the surrounding normal tissues in seven different forms of cancer, researchers observed a significant resemblance in bacterial composition between tumor tissue and normal adjacent tissue (NAT) Consequently, they have posited that intra-tumor bacteria may originate from NAT (Nejman et al., 2020). The disruption of the mucosal barrier due to numerous reasons may facilitate the infiltration of microorganisms into tumors. Research indicated that the tumor-associated bacteria in PDAC were moved from the gut to the pancreatic duct, and the microenvironment of this adenocarcinoma may enhance sensitivity to bacterial translocation (Leinwand and Miller, 2020). Furthermore, an investigation revealed that *Fusobacterium nucleatum* (Fn) may colonize colorectal cancer (CRC) by hematogenous dissemination. In this process, lectin Fap2 is crucial, binding to Gal-GalNAc present in CRC (Abed et al., 2016).

## 3 Functional mechanisms of the intratumoral microbiota

Intratumoral bacteria mostly inhabit cancer cells and immune cells, and microbial compositions vary across tumor types (Nejman et al., 2020). Previous studies have shown that the intratumor microbiota can contribute to cancer formation, progression and prognosis (Garrett, 2015; Sepich-Poore et al., 2021; Nejman et al., 2020). Here, we concentrate on emerging findings from recent years on the methods by which the intratumor microbiota promotes cancer growth (Figure 2), include the alteration of genetic material, activation of carcinogenic pathways, modification of anticancer medication metabolism, and facilitation of distant metastases.

### 3.1 Altering genetic material

Mutations generated by DNA damage significantly contribute to the beginning and advance of cancer. Oncoviruses have a significant role in the start of more than 10% of human malignancies (Khan and Hashim, 2014). Several studies have demonstrated that tumor viruses cause cancer via various direct and indirect mechanisms (Li et al., 2022). Oncoviruses, including hepatitis B virus (HBV) and human papillomavirus (HPV), induce cancer by incorporating their viral genomes into the host chromosome, resulting in alterations to host cells that lead to uncontrolled division and eventual malignancy. Certain carcinogenic bacteria may damage host DNA via various mechanisms, leading to genetic alterations that promote tumorigenesis (Costanzo et al., 2023). According to research, HPV stabilizes SMAD4 and thereby controls the host DNA damage repair, hence promoting viral replication and head and neck carcinogenesis (Citro et al., 2022). Research progress over the past four decades has profoundly revealed the carcinogenic mechanisms of oncogenic viruses (such as HPV and HBV): the oncoproteins they encode target and inhibit tumor suppressor proteins retinoblastoma protein (pRb) and tumor protein p53 (p53), disrupting cell cycle regulation and genomic stability, thereby driving malignant transformation (Moore and Chang, 2010, Levine, 2009, Weinberg, 1995, Munger and White, 2024). A recent study found that DNA tumor virus oncoproteins are powerful and selective inhibitors of the DNA-activated antiviral response (Lau et al., 2015). The HPV16 E7 oncoprotein directly suppresses the cyclic GMP-AMP synthase (cGAS)-stimulator of interferon genes (STING) signaling pathway, significantly reducing the expression of genes that encode type I interferon and pro-inflammatory proteins, hence facilitating immune evasion in several HPV-related tumors in murine models (Luo et al., 2020). The retrovirus known as Human T-lymphotropic virus type 1 (HTLV-1), which causes adult T cell leukemia, blocks DNA repair via the HTLV-1 Tax protein, which leads to genomic instability and the accumulation of carcinogenic mutations (Giam and Semmes, 2016). In 2020, a study found that polyketide synthase-positive *Escherichia coli* (pks + *E. coli*) can trigger genetic alterations in CRC cells (Pleguezuelos-Manzano et al., 2020). A research shows that adherent pathogenic bacteria, including Enteropathogenic *E. coli* and Enterohemorrhagic *E. coli*, capable of inducing transient diarrhea, connect with intestinal epithelial cells through their type 3 secretion system and deliver genotoxin-UshA, which breaks down the DNA of these cells, leading to cancer development (Liu Y. et al., 2022). Fn enhances esophageal squamous cell carcinoma (ESCC) development and chemical resistance by increasing the production of chemotherapy-induced senescence-associated secretory phenotypes through the activation of the DNA damage response system (Zhang J. W. et al., 2023). Fn infection promotes oral squamous cell cancer via inducing DNA double-strand breaks (DSBs) reliant on the Ku70/p53 pathway (Geng et al., 2020). Intestinal mucosal examination of individuals with familial adenomatosis revealed an abundance of *E. coli* and *Bacteroides fragilis*, which colonize epithelial cells and generate interlukin-17 (IL-17), causing extensive DNA damage to the epithelium (Dejea et al., 2018). *Bacteroides fragilis* toxin (BFT), released by *Bacteroides Fragilis*, degrades E-cadherin, causing changes in signaling pathways that induce the overexpression of spermidine oxidase, hence exacerbating irreparable DNA damage and potentially culminating in cancer (Cheng et al., 2020). Apart from direct DNA damage, BFT can generate significant concentrations of reactive oxygen species (ROS), which might thus adversely affect DNA. Long seen as connected to cancer is a rise in the ROS level. Higher degrees of ROS have been shown to be produced by several types of tumor cells than their normal counterparts, therefore resulting in damage to DNA, proteins, and lipids, hence inducing genomic disorder and DNA damage in cancer, which promotes genetic instability and carcinogenesis (Moloney and Cotter, 2018). Significantly, alongside BFT, *Enterococcus faecalis* has the capacity to produce ROS. ROS induce phosphorylated histone H2AX damages in human colon epithelial cells, hence triggering the DNA damage response system. This leads to G2 cell cycle arrest, chromosome dislocation, aneuploidy and tetraploidy, and ultimately DNA damage in the body (Wang et al., 2008). DNA damage in colonocytes can be mediated by reactive oxygen species/reactive nitrogen intermediates (ROS/RNI) created by inflammatory cells as well as endogenous ROS produced during the catabolism of microbial metabolites such as butyrate, both of which play major roles in inflammatory colon cancer models (Irrazabal et al., 2020). In addition, other modalities, including DNA methylation, non-coding RNAs, and histone modifications, constitute another important mechanism involved in cancer development through regulation of gene expression (Wen et al., 2023, Baba et al., 2023, Xu et al., 2022). Therefore, understanding the process of genetic material damage allows for preventative actions to postpone or minimize its onset, as well as early intervention and repair to preserve genomic integrity and lower the likelihood of cancer.

### 3.2 Activating carcinogenic pathways

The Wnt/β-catenin signaling pathway is a conserved and adaptable mechanism engaged in embryonic growth, tissue homeostasis, and many clinical illnesses. When this pathway is pathologically stimulated, β-catenin accumulates within the nucleus and facilitates the transcription of oncogenes like Cyclin D1 and c-Myc. Consequently, it facilitates the growth and development of various cancers, including ovarian, pancreatic, colorectal, hepatocellular, lung, and pancreatic cancers (Song et al., 2024, Wang H. et al., 2024). The Wnt signaling system depends on β-catenin, which is carefully regulated on three fronts: transcriptional activity, subcellular location, and protein stability (Shang et al., 2017). A study discovered that *Fusobacterium* adhesin A (FadA), produced by Fn, binds to E-cadherin on CRC and non-CRC cells, enhancing Fn adherence and invasion of epithelial cells. FadA increases β-catenin signaling and alters E-cadherin, resulting in increased production of transcription factors, oncogenes, Wnt genes, and inflammatory genes. In addition, it promotes CRC cell growth. This work identifies FadA as a potential diagnostic and therapeutic target for CRC and explains how Fn contributes to the disease’s development (Rubinstein et al., 2013). Apart from the FadA synthesized by Fn, which has the ability to initiate the Wnt/β-catenin pathway, another protein that can do so is the cytotoxin-associated gene A (CagA) protein. The cag pathogenicity island genes encode an antigenic effector protein as well as proteins that constitute a type IV bacterial secretion system that transports CagA from adhering *Helicobacter pylori* to host cells (Odenbreit et al., 2000, Fischer et al., 2001, Kwok et al., 2007, Shaffer et al., 2011). Unmodified CagA promotes β-catenin, upregulating cancer-related genes (Nagy et al., 2011). *Salmonella* secretes avirulence protein A (AvrA), which activates the β-catenin signaling system, promoting colon cancer (Lu et al., 2014, Lu et al., 2012). *B. fragilis* produces BFT, which stimulates E-calmodulin cleavage and activates β-catenin, leading to colon cancer (Sears, 2009). Trimethylamine-N-oxide is an intestinal microbe-dependent metabolite that binds to the farnesol X receptor (FXR), inhibiting the FXR-FGF15 axis and activating the Wnt/β-catenin signaling pathway (Zhang et al., 2024). A study discloses the mechanism via which Fn regulates microRNA21 (miR-21). Infection with Fn induces the toll-like receptors 4 (TLR4)/myeloid differentiation factor 8 (MYD88) and activates Nuclear Factor kappa-light-chain-enhancer of activated B cells (NF-κB) over time. Hyperactive NF-κB interacts to the miR-21, increasing its transcription in tumor tissues. The results revealed that Fn is substantially concentrated in cancer tissues, particularly in advanced tumors, resulting in increased miR-21 expression in locally progressed cancers. Furthermore, a subset of CRC patients with high Fn levels and miR-21 expression is more likely to have poor clinical outcomes (Yang et al., 2017). Furthermore, Intracellular *Porphyromonas gingivalis* stimulates CRC cell proliferation by stimulating the mitogen-activated protein kinase (MAPK)/extracellular regulated kinase (ERK) signaling pathway (Mu et al., 2020). The deleterious protein BFT-1 is excreted by enterotoxigenic *Bacteroides fragilis* (ETBF), which directly interacts with and stabilizes the Nucleotide binding oligomerization domain containing 1 (NOD1) protein. NOD1 is significantly expressed in ALDH^+^ breast cancer stem cells and works in conjunction with cyclin G-associated kinase (GAK) to phosphorylate and facilitate the lysosomal degradation of NUMB. This procedure increases the NOTCH1/HEY1 signaling pathway, hence enhancing the ability to foster stemness and chemoresistance in breast cancer stem cells (Ma W. et al., 2024). In brief, the tumor microenvironment can promote tumorigenesis by modulating cell signaling pathways. More study is needed in the future to investigate the various tumor microenvironment-mediated signaling pathways throughout carcinogenesis and development to provide accurate and successful treatments for cancer.

### 3.3 Modifying anticancer drug metabolism

According to one study, bacteria in pancreatic ductal adenocarcinoma (PDAC) may affect tumor susceptibility to gemcitabine. *Gammaproteobacteria*, which synthesize an elongated version of the bacterial enzyme cytidine deaminase (CDD_L_), may transform the chemotherapeutic agent gemcitabine (2′,2′-difluorodeoxycytidine) into its inactive derivative, 2′,2′-difluorodeoxyuridine (Geller et al., 2017). Genomic investigations conducted worldwide consistently show that the bacterial species Fn is more abundant in CRC than in non-cancerous colon tissues (Kostic et al., 2012). The research found that Fn infection lowered the chemical sensitivity of CRC cells to 5-fluorouracil (5-Fu) by upregulating BIRC3 both *in vitro* and *in vivo*, and that elevated Fn levels were associated with chemical resistance in advanced CRC patients receiving standard 5-Fu-based adjuvant chemotherapy after radical resection. Research suggests that BIRC3 and Fn might be used as therapeutic targets to reduce chemical resistance to 5-Fu treatment in advanced CRC (Zhang et al., 2019). Recent research indicates that Fn infection enhanced hsa_circ_0004085 production by hnRNPL and packed hsa_circ_0004085 into exosomes via hnRNP A1. Exosomes released by Fn-infected CRC cells transport hsa_circ_0004085 between them. Hsa_circ_0004085 reduces endoplasmic reticulum stress in recipient cells by regulating GRP78 and ATF6p50, resulting in resistance to oxaliplatin and 5-Fu (Hui et al., 2024). Fn has been found in CRC patients to have a role in oxaliplatin chemoresistance by activating the innate immune system. Data indicate that enhanced autophagy, facilitated by the downregulation of microRNA (miR-4802 and miR-18a-3p), resulted in oxaliplatin resistance *in vitro* (Yu et al., 2017). Research indicates that many bacterial species are involved in anthracycline metabolism, including *Streptomyces* WAC04685 and *Raoultella planticola*, which have been shown to inactivate doxorubicin via deglycosylation (Westman et al., 2012, Yan et al., 2018).

### 3.4 Assisting in distant metastasis

Intratumoral microbiota may affect the internal characteristics of oncocytes as well as their exterior surroundings, hence promoting cancer spread (Fu et al., 2023). A recent study investigated the functional importance of these intratumoral microbiota, largely utilizing the mouse spontaneous breast tumor model. They discovered that removing intratumoral microbiota dramatically decreased lung metastasis while without influencing main tumor development. During metastatic colonization, intratumoral bacteria carried by circulating tumor cells improved host-cell survival by reorganizing the actin cytoskeleton, increasing tolerance to fluid shear stress. The data indicate that tumor-resident bacteria, despite their modest biomass, play an essential role in driving cancer spread (Fu et al., 2022). ETBF, which produces BFT, colonizes both the mammary gland and the intestine, causing rapid epithelial hyperplasia. In contrast to non-toxigenic *Bacteroides fragilis*, ETBF colonization of the gut or breast ducts greatly promotes tumor cell proliferation and metastatic development (Parida et al., 2021). Exosomes from Fn-infected colonic cancer cells transfer miR-1246/92b-3p/27a-3p and CXCL16/RhoA/IL-8 from Fn-infected cells to uninfected cells, improving the cells’ capacity to migrate *in vitro* and encouraging tumor metastasis *in vivo* (Guo et al., 2020). *E. coli* residing inside tumors compromises the gut vascular barrier, a structural element regulating bacterial distribution along the gut-liver axis, influenced by the virulence regulator. Bacteria spread to the liver with gut vascular barrier impairment, promoting the formation of a premetastatic niche and the attraction of metastatic cells (Bertocchi et al., 2021). In addition, butyrate produced by *Roseburia* promotes tumor cell migration and invasion by enhancing M2 macrophages (Ma W. et al., 2024).

## 4 Therapeutic potential of the intratumoral microbiota in tumors

Chemotherapy and immunotherapy are now the most often used techniques of tumor treatment. Chemotherapy is delivered in the form of genotoxic medicines that damage the DNA of existing tumor cells while inhibiting the synthesis of new DNA during growth (Li, 2024). Immunotherapy works largely by blocking immunological checkpoints. Programmed cell death ligand 1 (PD-L1) suppresses T-cell proliferation by interacting with programmed cell death protein 1 (PD-1) on T-cell membranes, hence decreasing the body’s anti-tumor immune response (Zhou et al., 2024). Currently undergoing Phase 1 clinical trial evaluation (ClinicalTrials.gov Identifier: NCT04167137) in patients with advanced solid tumors and lymphomas, the engineered bacterial strain SYNB1891 represents a novel approach to targeting the tumor microenvironment. Designed to specifically activate the STING pathway within phagocytic antigen-presenting cells while simultaneously engaging complementary innate immune pathways, SYNB1891 highlights the therapeutic potential of engineered microbiota in oncology. This innovative strategy underscores the growing focus on microbiota-based interventions to precisely modulate antitumor immune responses (Leventhal et al., 2020). Emerging evidence highlights the multifaceted therapeutic implications of intratumoral microbiota, spanning from its diagnostic utility as a tumor-specific biomarker to its synergistic interplay with immunotherapies, with particular promise emerging through engineered bacterial interventions that precisely modulate the tumor microenvironment.

### 4.1 Intratumoral microbiota as a prospective biomarker for tumor diagnosis

The tumor microbiome, with its abundance characteristics and specific distribution in different tumor types and subtypes, shows great potential as a diagnostic and prognostic marker (Table 1). Recent studies have increasingly highlighted the diagnostic value of the tumor microbiome. Familial adenomatous polyposis is a precancerous condition linked to CRC. The detection of colibactin and BFT in colonic mucosa may serve as a promising biomarker for early CRC screening and risk stratification (Dejea et al., 2018). Furthermore, intratumoral microorganisms are essential to the prognostic significance of malignancies. A study found that elevated levels of Fn within tumors are closely associated with advanced tumor staging and poor prognosis. Specifically, in patients with ESCC, high levels of Fn in tumor tissue were not only significantly associated with shorter recurrence-free survival (RFS) but also predicted poorer response to neoadjuvant chemotherapy (Yamamura et al., 2019). The tumor-associated microbiome may also be associated with the prognosis of papillary thyroid carcinoma (PTC) subtypes (Gnanasekar et al., 2021). Another study on PTC subtypes described important similarities and differences in the fungal composition of PTC tumors based on PTC subtypes and identified associations with prognostic variables (John et al., 2023). The diversity of the tumor microbiome has a significant impact on the survival of patients with PDAC. The unique tumor microbiome composition observed in long-term survivors may contribute to the formation of a favorable tumor microenvironment, particularly through the presence of *Saccharopolyspora*, *Pseudoxanthomonas*, *Streptomyces*, and *Bacillus clausii* (Riquelme et al., 2019). A study has for the first time established tumor-derived microbial subtypes in pancreatic cancer, indicating that the microbiome is closely associated with the prognosis of pancreatic cancer patients (Zhang B. et al., 2023). By identifying microbial subtypes associated with gastric cancer prognosis and the efficacy of chemotherapy and immunotherapy, this study revealed the association between the tumor microbiome and clinical outcomes in gastric cancer. Among these, subtypes dominated by *Sphingobium*, *Delftia*, *Comamonas*, and *Stenotrophomonas* were associated with poorer overall survival (OS) (Wang H. et al., 2024). A study classified hepatocellular carcinoma (HCC) into distinct hepatic subtypes by clustering the relative abundance of door-level taxonomic units through analysis of the overall microbial composition of tumor samples. The results showed that even after adjusting for common prognostic factors (such as tumor differentiation, tumor size, tumor number, and major vessel invasion), these liver subtypes still demonstrated significant prognostic value. Additionally, the presence of low levels of *Akkermansia* and *Methylobacterium* was closely associated with poorer OS and RFS in patients (Sun et al., 2023). In a microbiome analysis of head and neck squamous cell carcinoma, high abundance of *Leptotrichia*, *Campylobacter*, and *Capnocytophaga* was significantly associated with improved overall survival, while high abundance of *Lactobacillus* was closely associated with decreased OS (Hamada et al., 2023). The composition and abundance of tumor-associated microbiomes significantly influence the prognosis, treatment response, and tumor microenvironment of patients with various cancers. This provides potential novel biomarkers for early screening, personalized treatment, and prognosis assessment in cancer. Researchers often classify tumor-associated microbiomes into different subtypes. By identifying specific microbial subtypes, it is possible to more accurately predict high-risk populations and assist in early diagnosis. Additionally, microbial subtypes are significantly associated with tumor staging, recurrence risk, and survival, aiding in patient stratification and guiding individualized follow-up strategies.

**TABLE 1:** Intratumoral microbiota as a prospective biomarker for tumor diagnosis.

Microbiota	Tumor type	Function and clinical implications	References
ETBF, pks + *E. coli*	Colorectal cancer	Elevated levels of interleukin-17, DNA damage in colon epithelial cells, faster tumor growth, and higher mortality rates	(Dejea et al., 2018)
Fn	Esophageal squamous cell carcinoma	Poor progression-free survival, poor response to neoadjuvant chemotherapy, and high tumor recurrence rate	(Yamamura et al., 2019)
*Frankia* sp.,*Trueperella pyogenes*	Papillary thyroid carcinoma	Better prognosis predicted by MACIS (distant metastases, patient age, resection completeness, local invasion and tumor size)	(Gnanasekar et al., 2021)
*Micrococcus luteus, Bradyrhizobium* sp.	Papillary thyroid carcinoma	Predicting poor prognosis using MACIS	(Gnanasekar et al., 2021)
*Candida albicans, Eremascus albus, Thanatephorusc ucmeris*	Papillary thyroid carcinoma	Predicting poorer prognosis by vital status, perineural invasion, and pathological staging	(John et al., 2023)
*Sachharopolyspora, Pseudoxanthomonas, Streptomyces*	Pancreatic ductal adenocarcinoma	Significantly improved patient prognosis through immune activation and metabolic regulation functions	(Riquelme et al., 2019)
*Sphingobium, Delftia, Comamonas, Stenotrophomonas*	Gastric cancer	Through pathways such as the bacterial secretion system, glutathione metabolism, and immune-suppressive microenvironment, treatment sensitivity is reduced and tumor progression is promoted, leading to poorer overall survival	(Riquelme et al., 2019)
*Akkermansi*a, *Methylobacterium*	Hepatocellular carcinoma	Low levels of Ackermannia or Methylobacterium were found to be associated with a poor long-term prognosis by the clinical features of degree of tumor differentiation, tumor size, macrovascular invasion and tumor number	(Wang H. et al., 2024)
*Leptotrichia, Campylobacter, Capnocytophaga, Lactobacillus*	Head and neck squamous cell carcinoma	high abundance of Leptotrichia, Campylobacter, and Capnocytophaga was significantly associated with improved overall survival, while high abundance of Lactobacillus was closely associated with decreased overall survival	(Hamada et al., 2023)

### 4.2 Intratumor microbiota and immunotherapy

Intratumoral microbiota bi-directionally modulates tumor immunogenicity and anti-tumor immune responses through metabolites and immunomodulatory pathways.

A study showed that the tumor microbiome significantly impacts the survival of PDAC patients by modulating immune infiltration (e.g., CD8 + T cell recruitment and Treg reduction). Specific genera, such as *Saccharopolyspora*, *Pseudoxanthomonas*, and *Streptomyces*, are associated with immune activation. Additionally, gut microbiota can remodel the tumor microenvironment through translocation or fecal microbial transplantation, enhancing anti-tumor immunity (Riquelme et al., 2019). A preclinical study demonstrated that Bifidobacterium, a gut microbiota bacterium, enhances the efficacy of local anti-CD47 immunotherapy by preferentially colonizing tumor sites and activating the STING signaling pathway (Shi et al., 2020). Another preclinical study elucidated that the microbiota in melanoma drives immunostimulatory differentiation of mononuclear phagocytes through the STING/IFN-I signaling axis, activating the NK-DC interaction cascade. High-fiber diets or fecal transplantation can reconfigure this pathway, enhancing the efficacy of immune checkpoint blockade. Clinical cohort analyses confirmed that responding patients exhibited significant enrichment of the IFN-I-NK-DC molecular signature in a microbiota-dependent manner (Lam et al., 2021). A study using a mouse model revealed that *Lactobacillus* L168 modulates anti-colitis-associated CRC immunity through its metabolite indole lactic acid (ILA) via a dual mechanism: on one hand, ILA enhances H3K27ac modification and chromatin accessibility at the IL12a gene enhancer region in dendritic cells, promoting IL12a expression and thereby activating CD8 + T cell-mediated anti-tumor immunity; on the other hand, ILA reduces chromatin accessibility and epigenetic modifications of the Saa3 gene in CD8 + T cells, lowering cholesterol metabolism and enhancing the functionality of tumor-infiltrating CD8 + T cells (Zhang Q. et al., 2023). The regulation of tumor immunity by the intratumor microbiota is often diverse. Some microbiota activate the host immune system through metabolites and immunomodulatory pathways to enhance immune surveillance of tumors, while others help tumor cells evade the immune system by inducing immune tolerance or activating immunosuppressive pathways. A preclinical study elucidated that the microbiota of PDAC shapes the immunosuppressive features of the tumor by modulating the immune microenvironment (myeloid-derived suppressor cells, macrophages, and T-cells) and activating the Toll-like receptor signaling pathway. In contrast, targeted microbial clearance strategies reversed this immunosuppression, including a decrease in myeloid-derived suppressor cells and an increase in M1 macrophage differentiation, which promoted CD4 + T cell differentiation toward Th1 and CD8 + T cell activation. In addition, bacterial clearance enhanced the efficacy of checkpoint immunotherapy, suggesting that the microbiota has potential as a therapeutic target for PDAC (Pushalkar et al., 2018). Another study revealed that Fn, through its surface protein Fap2, directly binds to the immunosuppressive receptor TIGIT and inhibits the anti-tumor activity of natural killer (NK) and T cells, thus helping tumors evade immune attack (Gur et al., 2015). Fn also downregulates miR-1322 through the NF-κB signaling pathway, activating the CCL20/M2 macrophage axis to promote CRC metastasis (Xu et al., 2021). Although the role of the tumor microbiome in regulating antitumor immunity has gradually been revealed, its clinical application still faces numerous challenges while also opening up new research directions for cancer treatment. First, the tumor-associated microbiome exhibits significant heterogeneity, including inter-tumor (e.g., differences in microbial composition between melanoma and CRC), intra-tumor (e.g., spatial distribution between tumor core and periphery), and inter-individual (influenced by host genetic background, dietary habits, and environmental factors) diversity, which complicates mechanism elucidation and precision intervention (Wang et al., 2022, Yu et al., 2022, Simpson et al., 2022). Second, the interaction between the microbiome and the immune system involves multidimensional networks, including metabolites (such as short-chain fatty acids and indole derivatives), epigenetic modifications, and signaling pathways, whose dynamic regulatory mechanisms remain poorly understood (Zhang Q. et al., 2023, Mann et al., 2024, Moratiel-Pellitero et al., 2024). Additionally, existing technologies have limitations, such as the difficulty of detecting low-abundance microbial communities using 16S rRNA sequencing and challenges in functional validation using single-cell RNA sequencing (Lloréns-Rico et al., 2022, Zhao et al., 2021). Finally, the safety and efficacy of microbiota intervention strategies (such as probiotics and selective antibiotic removal) need to be further validated in large-scale clinical trials to ensure their reproducibility and universality in clinical practice (Wang H. et al., 2024; Chen L. et al., 2023). These challenges point the way forward for future research, including the development of high-resolution technologies to analyze microbial spatial distribution, the construction of multidimensional interaction network models, and the optimization of personalized design of microbiota intervention strategies.

### 4.3 Engineering bacteria

Traditional tumor therapies have several limitations, and patients frequently do not have a better prognosis. Engineered bacteria are bacteria that have been genetically manipulated to provide certain functionalities that can be used in tumor treatment. These bacteria can be designed to thrive and replicate in the tumor microenvironment while also expressing certain genes. The colonization of intratumoural microorganisms provides new methods for anticancer therapy. The disadvantages of standard anti-cancer medications can be overcome by employing gut microbiota as *in vivo* delivery methods (Liang et al., 2019). As a result, the change of tumor microorganisms into synthetic bacteria opens up new possibilities for tumor therapy. Chen et al. successfully overcame both challenges Engineered *S. epidermidis* colonizes tumors and induces tumor-specific T cells to multiply, penetrate local and metastatic lesions, and produce cytokines (Chen L. et al., 2023). Canale et al. created a modified probiotic *E. coli* strain that colonizes tumors and continually converts ammonia, a metabolic waste product found in tumors, to L-arginine. Tumor colonization with these bacteria enhanced intra-tumor L-arginine concentrations as well as the number of T lymphocytes invading the tumor (Canale et al., 2021). A live therapeutic platform based on genetically modified *Bacillus thuringiensis* spores, which achieves dual functions of near-infrared photothermal antitumor activity and broad-spectrum reactive oxygen and nitrogen species (RONS) clearance through continuous synthesis of melanin. RONS include hydrogen peroxide (H_2_O_2_), hydroxyl radicals (•OH), superoxide anions (O_2_•^–^), nitric oxide (•NO), and peroxynitrite radical (ONOO^–^). Concurrently, it induces tumor cell apoptosis and inhibits pro-inflammatory factors (TNF-α/IL-6), providing a microbe-photothermal-antioxidant tri-modal strategy for the synergistic treatment of inflammation-associated tumors. The use of genetically engineered microbes in cancer therapy has great potential (Chen M. et al., 2024). A study has developed an engineered bacterium targeting cysteine metabolism using synthetic biology. This bacterium can colonize the tumor microenvironment of PDAC and continuously express engineered cysteinease (CGL) under hypoxic conditions, thereby efficiently consuming cysteine and completely blocking its metabolism. This strategy overcomes the limitations of traditional methods, such as insufficient targeting and compensatory metabolic escape, by destroying the iron death defense system of PDAC cells, inducing lipid peroxidation accumulation, and triggering potent iron death, thereby providing a novel microbial-based precision metabolic intervention strategy for PDAC treatment (Qiao et al., 2025). However, there are several difficulties. Bacteria are widely available, yet their unknown biological properties might have unintended consequences or therapeutic outcomes. For example, when bacteria colonize the human body, they can cause sickness. In reality, bacteria may not be limited to the cancer location and may not always be plentiful in the tumor, even if they can be designed to target tumors. Furthermore, they might circulate, increasing the toxicity *in vivo*. Second, while drugs can be precisely delivered to cancer sites *in vivo* utilizing engineered bacteria as carriers, this remains a challenge.

## 5 Future perspectives and challenges

### 5.1 Challenges in the detection of intratumoral microbes

One of the key problems in microbial detection within tumors is low microbial biomass, which is frequently low in cancer tissues and makes identification more difficult. Furthermore, the high ratio of host genome to microbial genome in cancer samples causes considerable host genome contamination, making it difficult to identify microbial communities (Liu W. et al., 2023, Fang et al., 2025). Furthermore, samples are easily contaminated by the outside environment during collection, transit, and storage, which may provide challenges for microbial community study (Liang et al., 2023). The complexity of the microbial communities seen in tumors makes data processing challenging. Accurate microbial community analysis and interpretation need the use of appropriate methodologies and equipment. Given the magnitude of the difficulties at hand, it is worthwhile to study a variety of options, including improved ways for handling and maintaining samples, the use of more complex detection technologies, and the development of more powerful data processing tools.

### 5.2 Challenges of clinical translation of tumor microbes

Tumor-microbe interactions research is experiencing a paradigm shift from correlation to causation, the core of which lies in systematically resolving the molecular mechanisms of key functional bacteria (e.g., Clostridium nucleatum promotes tumor metastasis through activation of the Wnt/β-catenin pathway by the FadA adhesin) by means of multi-omics integration technologies (e.g., single-cell sequencing, spatial transcriptomics), and utilizing synthetic biology (e.g., bionanobacterial encapsulation, CRISPRi dynamic regulation) to enhance the colonization efficiency and therapeutic controllability of engineered bacteria in the tumor microenvironment (Li et al., 2023). At the same time, AI-driven prediction models will be constructed by combining individualized microbiome characteristics (e.g., enterotyping, HLA polymorphisms, and baseline bacterial flora composition) to guide precise therapeutic strategies (Mkilima, 2025; Sternes et al., 2020; Zhu et al., 2024). In this way, we can systematically solve the key bottlenecks in the mechanism analysis, therapeutic stability and individualized application of microbial therapies, and promote their advancement from laboratory research to clinical translation, thus opening up a whole new dimension for tumor immunotherapy.

### 5.3 The prospects and limitations of tumor microbiology therapy

Microbial-based therapies, including oncolytic viruses and bacteria, have demonstrated unique potential in cancer treatment due to their ability to specifically target tumors, induce immune responses, and enhance the efficacy of conventional therapies (Zhou et al., 2018; Canale et al., 2021; Liu L. et al., 2023). However, their clinical application still faces significant safety challenges: while oncolytic virus therapies generally have a favorable safety record, with very few adverse events directly attributed to viral replication in clinical trials, concerns remain regarding off-target effects and the risk of viral mutations. Systemic administration of oncolytic viruses is susceptible to neutralization by antibodies and may lead to hepatotoxicity (Zhao et al., 2024; Chang et al., 2009). On the other hand, although robust bacterial colonization within tumors can induce antitumor immunity, it often accompanies dose-limiting inflammatory responses (e.g., abscess formation) (Toso et al., 2002). Abscess formation is clearly an ideal therapeutic outcome but also a significant toxicity. Furthermore, the risk of viral mutations and bacterial dissemination to normal tissues further limits the balance between efficacy and safety. There is an urgent need to precisely regulate microbial activity through genetic engineering and optimize delivery strategies to ensure tumor-specific killing while minimizing systemic toxicity.

### 5.4 Future outlook

More than a century ago, Dr. William Coley proposed that all types of malignant tumors may be influenced by the involvement of microorganisms (Coley, 1910). However, systematic verification began with the application of high-throughput sequencing technology in the early 21st century. In 2020, Nejman et al. used 16S rRNA sequencing to systematically confirm for the first time the existence of specific microbial communities in solid tumors such as pancreatic cancer and breast cancer, and that most of the bacteria in tumors were found in cancer cells and immune cells (Nejman et al., 2020). However, their data cannot determine whether bacteria within tumors play a causal role in cancer development. In terms of mechanism research, Bullman et al. revealed the correlation between microorganisms and tumor metastasis through clinical evidence, showing that the abundance of Fn in colorectal cancer is significantly positively correlated with the risk of liver metastasis (Bullman et al., 2017). In 2013, Rubinstein et al. confirmed that Clostridium difficile activates the β-catenin pathway through the FadA adhesion (Rubinstein et al., 2013). In 2015, Gur et al. revealed that tumors utilize the Fap2 protein of Fn to inhibit immune cell activity through TIGIT (Gur et al., 2015). In 2022, Liu et al. systematically explored the CRC-associated microbiome, demonstrating the applicability of multi-domain and multifunctional markers as diagnostic tools for colorectal cancer, as well as their potential as therapeutic targets for colorectal cancer treatment (Liu N. N. et al., 2022).

Current understanding of the interaction between tumor-associated microbiota and tumor cells remains at a relatively basic level. Complex animal models can simulate more realistic physiological and pathological environments in humans, thereby providing richer and more reliable evidence for preclinical research. For example, by constructing animal models carrying specific gene mutations and infected with specific microorganisms, we can closely observe changes in tumor cell proliferation and metastasis under microbial stimulation, thereby clarifying the specific mechanisms by which microorganisms influence tumor development at various stages. The application of interdisciplinary methods is indispensable in tumor-associated microbiome research. The relationship between tumor-associated microbiomes and tumor formation and development is a complex system involving multiple disciplines such as microbiology, oncology, and bioinformatics. We need to utilize bioinformatics technologies to analyze and mine massive microbiome data, and employ mathematical models to quantitatively describe the interaction intensity and dynamic processes between microorganisms and tumor cells. Through interdisciplinary collaboration, we can gain a more comprehensive and in-depth understanding of the mechanisms by which tumor-associated microbiota influence tumor development, providing scientific basis for future research and treatment. In the field of antitumor therapy, studying the relationship between identified microbial characteristics and tumor response regulation is of great significance. The characteristics of tumor-associated microbiota may influence tumor response to treatment. By deeply exploring this relationship, we can identify new clinical intervention targets. Based on these targets, we can develop more targeted treatment strategies to enhance the efficacy of tumor therapy. Exploring combined treatment strategies based on microbial intervention is also a highly promising research direction. Single treatment methods often struggle to completely suppress tumor growth and metastasis, but combining microbial intervention with traditional methods such as surgery, chemotherapy, radiotherapy, and immunotherapy may produce synergistic effects, enhancing treatment efficacy and improving treatment outcomes for cancer patients.

## 6 Integrating reviews and emerging technologies in intratumoral microbiome research

### 6.1 Research on the microbiome characteristics of different malignant tumors

The current reviews exhibit a significant bias toward specific cancer types, with a strong focus on gastrointestinal tumors (particularly CRC), while research on non-gastrointestinal cancer types remains relatively limited. Taking CRC as an example, Li et al. systematically outlined the carcinogenic mechanisms of key microorganisms: Fn and pks^+^ *E. coli* induce DNA damage, promote immune evasion, and activate carcinogenic signaling pathways to drive tumorigenesis (Li S. et al., 2021). Notably, this review also explores the mechanisms by which the microbiome influences treatment response. For example, Fn modulates autophagy-associated proteins to reduce the sensitivity of tumor cells to oxaliplatin (Yu et al., 2017). Regarding interventional strategies, beyond conventional antibiotics, the study summarizes two novel approaches: the M13@Ag complex—formed through electrostatic assembly of the M13 phage protein capsid and inorganic silver nanoparticles—which specifically eliminates Fn and remodels the tumor immune microenvironment (Dong et al., 2020); and natural product interventions, such as zerumbone (derived from *Zingiber zerumbet*), which inhibits ETBF-induced intestinal inflammation-associated CRC progression by modulating IL-17, β-catenin, Stat3, and NF-κB pathways (Hwang et al., 2020).

In the field of gastric cancer research, Liu et al.’s review innovatively reveals the dynamic evolution of gastric microbiota composition and function from healthy states to distinct stages of gastric carcinogenesis (Liu et al., 2024); this study further elucidates how interactions between *Helicobacter pylori* and commensal bacteria modulate chronic inflammation to influence gastric cancer development, specifically demonstrating that the *Weizmannia coagulans* strain BCF-01—isolated from healthy gastric mucosa—exhibits significant anti-*H. pylori* activity. This strain effectively alleviates *H. pylori*-induced gastric dysbiosis by modulating microbial composition and attenuates post-infection inflammatory responses (Chen Z. et al., 2024).

In contrast, research on HCC remains relatively preliminary. An exploratory study published on July 10, 2025, revealed that liver tumor tissues harbor microbiota predominantly originating from the upper gastrointestinal tract—including *Streptococcus* spp., *Gemella haemolysans*, and *H. pylori*. Microbial diversity showed a positive correlation with lesion count and was associated with poor prognosis. These microorganisms may translocate to the liver via the biliary system or portal vein, though their precise mechanistic roles in hepatocarcinogenesis require further investigation (Schulz et al., 2025).

### 6.2 Treatment strategy conversion direction

The current review proposes a variety of therapeutic strategies targeting the tumor microbiome, which can be summarized into three main approaches:

The use of selective antibiotics to eradicate specific procancer microbiota represents the most direct interventional strategy. Through systematic review of preclinical and clinical data, Nardo et al. analyzed synergistic mechanisms between commonly used antibiotics and standard chemotherapy, tyrosine kinase inhibitors (TKIs), and immunotherapy (Nardo et al., 2024): in genitourinary cancer models, ciprofloxacin demonstrated significant synergy with chemotherapeutic agents (El-Rayes et al., 2002; Engeler et al., 2012); Chen et al. further showed that lymacycline—a tetracycline derivative—reverses resistance to the EGFR-TKI icotinib in lung cancer cells by inhibiting EGFR phosphorylation and blocking GRB2-mediated AKT/ERK/STAT3 signaling (Chen et al., 2020). Beyond direct anticancer effects, multiple preclinical studies reveal antibiotics can enhance immune responses, exemplified by doxycycline increasing tumor cell MHC-I expression through autophagy inhibition (overcoming an immune evasion mechanism), thereby establishing a rationale for combination immunotherapy (Xiao et al., 2023). The review additionally catalogs antibiotics currently applied in clinical trials, including clarithromycin, doxycycline, metronidazole, ciprofloxacin, azithromycin, levofloxacin, and tigecycline.

Supplementing beneficial microbiota or modulating microbial composition constitutes another critical interventional strategy. Multiple reviews confirm that probiotics play key roles in treating various cancers—including breast, skin, and colorectal carcinomas (Xie et al., 2022; Ridlon and Gaskins, 2024; Leinwand and Miller, 2020)—while highlighting safety concerns such as horizontal transfer of antibiotic resistance genes to gut microbiota, adverse drug reactions, and tolerability challenges (Morsli et al., 2025). Given that > 40% of chemotherapy patients develop intestinal dysbiosis (requiring oral live biotherapeutic products for mitigation), yet conventional probiotics are vulnerable to chemotherapeutic cytotoxicity (Morsli et al., 2025; Dahlgren et al., 2021), engineered probiotics have emerged as a priority development direction: Liu et al.’s “tea polyphenol-armored supra-particulate probiotics” (SupraLBT) significantly enhance probiotic survival in chemotherapy environments through self-assembled tea polyphenol-milk protein protective layers (Liu et al., 2025); addressing immunotherapy resistance caused by pancreatic cancer’s intratumoral microbiome dysbiosis, Han’s team constructed gallium-polyphenol network-encapsulated *Lactobacillus rhamnosus* GG (LGG@Ga-poly) to enhance efficacy by regulating microbiota-immune interactions (Han et al., 2024). Recent research further identifies the perioperative period as a golden window for probiotic intervention—administration of *Clostridium butyricum* CBM588 before/after colon resection shortens time to first flatus by 30% and increases circulating T-cell counts, advancing its role from adjuvant therapy to a core mediator of “gut restoration-immune activation-recurrence suppression” tumor ecological regulation (Yang et al., 2025).

Modulating the microbiome to enhance immunotherapy response represents a current research hotspot. A review indicates that specific microbial signatures can predict the efficacy of immune checkpoint inhibitors (ICI) (Verhaert and Aspeslagh, 2024); Lu et al. synthesized studies on gut microbiota’s impact on ICI outcomes and elucidated mechanisms by which microbiota interact with innate and adaptive immune cells to improve ICI efficacy (Lu et al., 2022). Multiple studies confirm significant associations between gut microbiota and ICI responsiveness in non-small cell lung cancer (NSCLC), hepatocellular carcinoma (HCC), and melanoma patients (Zheng et al., 2019, Jin et al., 2019, Matson et al., 2018). Subsequently, landmark clinical trials demonstrated that combining fecal microbiota transplantation (FMT) from ICI responders with anti-PD-1 therapy overcomes PD-1 blockade resistance in melanoma patients (Baruch et al., 2021). Recent research further revealed that the gut microbial metabolite formate activates the Nrf2 pathway within CD8^+^ T cells, enhancing their antitumor functionality and substantially boosting ICI efficacy (Phelps et al., 2025). Understanding the biological mechanisms through which gut microbiota and their metabolites regulate antitumor immunity and immunotherapy responses is crucial for rationally modulating microbial activity to improve ICI outcomes. Therapeutic strategies to enhance ICI efficacy via microbiota modulation—including FMT, probiotics, and engineered microbiomes (Baruch et al., 2021, Hibberd et al., 2017, Canale et al., 2021)—require future research to shift focus from correlative validation to causal mechanistic investigation, enabling precision enhancement of ICI effectiveness.

### 6.3 Methodology and technology application

Intratumoral microbiome research has emerged as a pivotal branch of tumor microenvironment investigation, with current reviews revealing significant technological evolution in detection methodologies. The contemporary methodological framework encompasses four major categories: sequencing-based techniques, microscopic imaging, culturomics, and immunological detection. Wang et al. systematically reviewed the advantages and limitations of emerging technologies (Wang N. et al., 2025): early studies relied on 16S rRNA sequencing for microbial composition analysis. While this method can decipher species diversity and evolutionary relationships (Nejman et al., 2020), its resolution for bacteria with minimal interspecies differences remains inadequate due to amplified region constraints (typically limited to V3–V4 hypervariable regions) (Zhang Q. et al., 2023). In contrast, 5R 16S rDNA sequencing achieves markedly enhanced resolution through full-length sequence coverage, enabling high-precision identification of microbial communities in breast tumors (Nejman et al., 2020). Given that 16S rRNA sequencing targets only known bacteria and cannot detect fungi/viruses, metagenomic sequencing captures all DNA in tumor samples (including microbial and host DNA), permitting microbial identification at species or strain levels (Xue et al., 2023). To address critical challenges of low microbial biomass, DNA degradation, and host contamination, 2bRAD microbiome sequencing (2bRAD-M) provides qualitative and quantitative results for bacteria, archaea, and fungi at species resolution (Sun et al., 2022). Single-cell transcriptomic profiling faces unique obstacles—conventional scRNA-seq depends on polyA primers to capture eukaryotic mRNA, whereas bacterial mRNA lacks poly(A) tails and exhibits low abundance (Xue et al., 2023, Sun et al., 2022). Innovative technologies overcome this bottleneck: PETRI-seq (prokaryotic expression profiling by tagging RNA *in situ* with combinatorial indexing) and MicroSPLiT (microbial split-pool ligation transcriptomics) resolve prokaryotic transcriptomes (Blattman et al., 2020, Kuchina et al., 2021); BacDrop and smRandom-seq achieve precise localization of microbe-host interaction subpopulations by efficient rRNA removal and mRNA enrichment (Xu et al., 2023, Ma et al., 2023). Furthermore, INVADEseq (invasion-adhesion-directed expression sequencing) identifies cell-associated bacteria and their host interactions in patient tumors, revealing transcriptional alterations in inflammation, metastasis, cellular dormancy, and DNA repair pathways (Galeano Niño et al., 2022). The SAHMI (single-cell analysis of host-microbiome interactions) computational pipeline systematically recovers and denoises microbial signals in clinical tissues, enabling tumor-microbiome interaction studies at single-cell resolution (Xu et al., 2025). Wang et al. further systematically reviewed advances in microscopic imaging techniques for intratumoral microorganisms: classical fluorescence *in situ* hybridization (FISH) employs fluorescent probes targeting bacterial 16S rRNA to detect microbial DNA/RNA (Xue et al., 2023), but exhibits insufficient signal intensity due to poor cell permeability and low nucleic acid copy numbers (Zwirglmaier, 2005). The novel RNAscope-FISH technology achieves single-molecule visualization within individual cells through signal amplification and background suppression, successfully resolving the spatially heterogeneous distribution of Fn in CRC (Galeano Niño et al., 2022, Wang et al., 2012). Additionally, the multiplexed rapid semi-quantitative technique PEHPSI (Prokaryotic-Eukaryotic Hybrid Probe *in Situ* Imaging) simultaneously characterizes bacteria and host cells (e.g., breast cancer subtypes and immune cells) (Feng et al., 2022). In ultrastructural imaging, transmission electron microscopy (TEM) reveals intact bacterial morphology and degradation contours within lysosomes of intrahepatic cholangiocarcinoma tumor cells (Chai et al., 2023). Correlative light-electron microscopy (CLEM) integrates fluorescence microscopy and electron microscopy, enabling high-specificity localization of intracellular bacteria in melanoma cells (Kalaora et al., 2021). For 3D imaging, tissue clearing technology achieves whole-tissue 3D visualization by minimizing light scattering (Lee et al., 2021). An innovative strategy combining tissue clearing, immunofluorescence labeling, light-sheet microscopy, and image processing allows direct, contamination-free observation of bacterial lipopolysaccharide (LPS) spatial distribution in gliomas (Zhao et al., 2022). Distinct from spectroscopic methods, CAST-R-HP technology integrates single-cell Raman spectroscopy (SCRS) with machine learning to accurately distinguish *H. pylori* drug-susceptibility phenotypes in biopsy specimens, supporting targeted sorting, low-bias whole-genome amplification, and mechanistic elucidation of phenotypic determinants (Liu Y. et al., 2022).

### 6.4 Emerging perspectives and development trends

The intratumoral microbiome field is undergoing a transformative paradigm shift driven by three key innovations: ecological management theories for dynamic microbiome regulation, multi-omics integration enabling precision interventions, and engineering technologies accelerating clinical translation.

In a June 2025 *Nature Reviews Clinical Oncology* publication, a review innovatively proposes integrating ecological principles into cancer patient microbiome management frameworks. This paradigm transcends traditional reductionist approaches—such as “beneficial/detrimental bacteria” dichotomies or simplified biodiversity metrics—by reconceptualizing the microbiome as a dynamic functional organ rather than a static biomarker. Through mathematical ecological models (e.g., ecological networks, survival analyses, neural network-based ODEs) processing longitudinal microbiome data, it predicts dysbiosis risks while leveraging ecosystem complexity and redundancy to optimize cancer therapeutics. Routine longitudinal monitoring via non-invasive methods (e.g., fecal 16S rRNA sequencing), integrated with these computational tools, enables: (1) complication risk prediction, (2) treatment regimen optimization (minimizing microbial disruption), and (3) precision intervention design (restoring impaired microbial functions). Ecology-guided interventions—including fecal microbiota transplantation (FMT), computationally engineered microbial consortia, phage therapy, and synthetic biology constructs—ultimately restore or maintain homeostatic microbiome states (Xavier, 2025).

The core direction of future research lies in integrating multi-omics data (encompassing genomics, transcriptomics, metabolomics, and immune response profiling) to systematically decipher host-microbiome interaction networks. Zhang et al. systematized the methodological value of genomics, transcriptomics, proteomics, and metabolomics in tumor microbiome research, establishing a standardized omics analysis toolbox (Zhang Q. et al., 2023). Through multi-tiered validation via 16S rDNA sequencing, metagenomic sequencing, and transcriptome sequencing, Qiao’s team demonstrated that intratumoral bacterial load serves as an independent prognostic indicator for nasopharyngeal carcinoma patients, providing risk-stratified guidance for therapeutic decisions across malignant progression risk levels (Qiao et al., 2022). Chang et al. further implemented artificial intelligence for multi-omics integration within the tumor immune microenvironment. By fusing genomic, transcriptomic, radiomic, and histopathological imaging data through deep learning and machine learning, they significantly enhanced biomarker discovery efficiency and accuracy, constructing multimodal predictive models to support personalized immunotherapy decision-making (Chang et al., 2025).

Wang et al. systematically analyzed how nanotechnology enhances microbial therapies for precision oncology. Through gene editing, metabolic pathway reprogramming, or surface molecule modification of bacterial strains, engineered microorganisms achieve highly selective colonization within tumor tissues (Wang R. et al., 2025). These engineered bacteria precisely express/release proteins, metabolites, and signaling molecules, significantly improving *in situ* imaging and localization accuracy (Cronin et al., 2012, Bourdeau et al., 2018, Jiang et al., 2013). Their intelligent lysis capability enables responsive release of antitumor drugs or immunomodulatory molecules into the tumor microenvironment upon external stimuli, establishing a targeted, efficient, and dynamically controllable drug delivery system (Gurbatri et al., 2020). As a breakthrough application, Liu’s team developed “tea polyphenol-armored supra-particulate probiotics” (SupraLBT), where the self-assembled tea polyphenol-milk protein protective layer increases the survival rate of probiotics during chemotherapy by 56 to 133 times. Additionally, oral administration of SupraLBT combined with chemotherapy (doxorubicin) resulted in tumor regression rates 2.35 times higher than those achieved with doxorubicin alone, offering an innovative solution to chemotherapy-induced intestinal dysbiosis.

## 7 Conclusion

This study systematically elucidates the biological role of bacteria in tumorigenesis and tumor progression, and explores the diagnostic and therapeutic potential of the tumor microflora. Based on an analysis of the core challenges and future research directions in this field, this study systematically analyzes existing reviews, integrates their research paradigms, methodologies, and key conclusions, and aims to stimulate innovative research paths in the field of cancer microbiology.
